# A New Hybrid δ-Lactone Induces Apoptosis and Potentiates Anticancer Activity of Taxol in HL-60 Human Leukemia Cells

**DOI:** 10.3390/molecules25071479

**Published:** 2020-03-25

**Authors:** Katarzyna Gach-Janczak, Joanna Drogosz-Stachowicz, Angelika Długosz-Pokorska, Rafał Jakubowski, Tomasz Janecki, Jacek Szymański, Anna Janecka

**Affiliations:** 1 Department of Biomolecular Chemistry, Medical University of Lodz, Mazowiecka 6/8, 92-215 Lodz, Poland; joanna.drogosz@umed.lodz.pl (J.D.-S.); angelika.dlugosz@umed.lodz.pl (A.D.-P.); anna.janecka@umed.lodz.pl (A.J.); 2 Institute of Organic Chemistry, Lodz University of Technology, Zeromskiego 116, 90-924 Lodz, Poland; rafalj@cbmm.lodz.pl (R.J.); tomasz.janecki@p.lodz.pl (T.J.); 3 Centre of Molecular and Macromolecular Studies, Polish Academy of Sciences, Sienkiewicza 112, 90-363 Lodz, Poland; 4 Central Laboratory, Medical University of Lodz, Mazowiecka 6/8, 92-215 Lodz, Poland; jacek.szymanski@umed.lodz.pl

**Keywords:** coumarins, α-methylidene-δ-lactones, cytotoxicity, apoptosis, leukemia, synergistic effect

## Abstract

In the search for new drug candidates, researchers turn to natural substances isolated from plants which may be either used directly or may serve as a source for chemical modifications. An interesting strategy in the design of novel anticancer agents is based on the conjugation of two or more biologically active structural motifs into one hybrid compound. In this study, we investigated the anticancer potential of 4-benzyl-5,7-dimethoxy-4-methyl-3-methylidene-3,4-dihydro-2*H*-chroman-2-one (DL-247), a new hybrid molecule combining a chroman-2-one skeleton with an *exo*-methylidene bond conjugated with a carbonyl group, in human myeloid leukemia HL-60 cell line. The cytotoxicity of the new compound was tested using MTT assay. The effect of DL-247 on cell proliferation and apoptosis induction were studied by flow cytometry, fluorometric assay and ELISA analysis. DL-247 displayed high cytotoxic activity (IC_50_ = 1.15 µM, after 24 h incubation), significantly inhibited cell proliferation and induced apoptosis by both, the intrinsic and extrinsic pathways. A combination of DL-247 with taxol exhibited a strong synergistic effect on DNA damage generation, apoptosis induction and inhibition of cell growth.

## 1. Introduction

Millions of new cancer cases are being diagnosed each year worldwide and this number is still increasing. In the search for modern anticancer drugs, scientists often turn their interests to compounds of natural origin. A large number of anti-cancer agents selected for clinical trials are plant-derived compounds [1].

**Figure 1:**
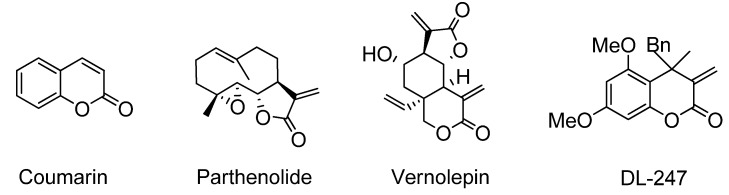
Structures of coumarin, parthenolide, vernolepin and a new hybrid DL-247 (4-benzyl-5,7-dimethoxy-4-methyl-3-methylidene-3,4-dihydro-2*H*-chroman-2-one).

Coumarins (chromen-2-ones) (Figure 1) and dihydrocoumarins (chroman-2-ones) are a large family of phytochemicals widely distributed in nature that recently have drawn much attention due to their diverse biological properties, including anti-inflammatory, antibacterial, antiviral, and antitumoral activities [2,3]. The cytotoxic effect of both natural coumarins and their synthetic derivatives was shown in various cancer cell lines, such as lung (A549, H727), renal (ACHN, breast (MCF7), leukemia (U-937 and HL-60) and in experimental animal models of cancer [2,4,5]. Coumarins exert their anticancer activity interacting with many enzymes and cellular proteins, which results in the cell cycle arrest, induction of apoptosis and inhibition of cell proliferation [3,6]. However, the molecular mechanisms involved in the anticancer effect of coumarins still remain to be fully elucidated.

Another interesting group of biologically important compounds of natural origin are molecules containing an *exo*-methylidene moiety conjugated with a carbonyl group [7]. Such substances are widely spread in plants of the *Compositae* family and exhibit, among others, anti-inflammatory and cytotoxic/anticancer activity [7,8]. Compounds with unsaturated lactone functionality react via the Michael-type reaction with biological nucleophiles, e.g., enzymes, proteins, and free intracellular glutathione, which leads to the formation of covalent adducts and disrupts key biological processes in the cells [9]. The most common in nature group of compounds with such motif are α- methylidene-γ-lactones, with parthenolide (PTL) (Figure 1), isolated from feverfew (*Tanacetum parthenium*) being the most studied representative [10]. Another structurally similar systems, less abundant in nature are α-methylidene-δ-lactones. Well know examples of such compounds are vernolepin (Figure 1) and vernomenin isolated from *Vernonia hymenolepis* [11] and crassin from *Pseudoplexaura porosa* [12] showing some cytotoxicity against human pharyngeal carcinoma (KB). Along with the increasing interest in α-methylene-γ-lactones, their homologues, α-methylene-δ-lactones, have also become frequent targets for both, the synthetic and biological studies. High cytotoxicity and anticancer properties of compounds with *exo*-methylidene moiety conjugated with a carbonyl group have been shown in several studies on numerous human cancer cell lines and also in animal models [7,8,13,14]. But so far, only one compound of this group, an arglabin derivative, dimethylaminoarglabin, has been registered and introduced into medical practice as an anticancer drug in Kazakhstan, Russia, and the USA (Georgia) [15].

An interesting approach in the design of novel anticancer agents is the synthesis of hybrid compounds combining two or more structural motifs with different biological functions. In our previous reports we described a few series of such synthetic hybrids with distinct pharmacophore units, a chromen-2-one skeleton and an α-methylidene-δ-lactone moiety [16,17]. Several of these compounds showed remarkable in vitro cytotocic activity against cancer cell lines. The most potent analogs were further evaluated as potential anticancer agents [18,19,20]. We have shown that 1-isopropyl-2-methylene-1,2-dihydrobenzochromen-3-one induced the intrinsic apoptotic pathway and suppressed cell migration and invasion in breast cancer MDA-MB-231 and MCF-7 cells [18]. Another compound, (R*)-8-methoxy-3-methylene-4-[(S*)-2-oxocyclohexyl]chroman-2-one inhibited cell proliferation and induced apoptosis in MCF-7 and HL-60 cells [19]. The apoptosis was related to DNA damage induction and decreased activity of proteins involved in DNA damage repair [20]. Recently, we obtained a new library of chroman-2-ones containing a conjugated *exo*-methylidene moiety. Several of these compounds showed strong cytotoxicity against leukemia (HL-60 and NALM-6) and breast cancer (MCF-7) cells [21]. In the present study, we evaluated the proapoptotic activity of one of these hybrids, 4-benzyl-5,7-dimethoxy-4-methyl-3-methylidene-3,4-dihydro-2*H*-chroman-2-one (DL-247) (Figure 1), and tested anticancer properties of this compound in combination with the selected anticancer drugs, 5-fluorouracil (5-FU), taxol (Tx), and oxaliplatin (Ox).

## 2. Results

### 2.1. Chemistry

**Scheme 1:**
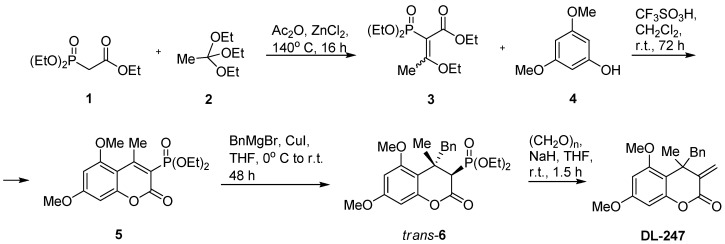
Synthesis of 4-benzyl-5,7-dimethoxy-4-methyl-3-methylidene-3,4-dihydro-2*H*-chroman-2-one (DL-247).

4-Benzyl-5,7-dimethoxy-4-methyl-3-methylidene-3,4-dihydro-2*H*-chroman-2-one (DL-247) was obtained in the reaction sequence shown in Scheme 1. Starting ethyl diethoxyphosphorylacetate (**1**) heated with triethylorthoacetate (**2**) in the presence of Ac_2_O and ZnCl_2_ as catalysts gave the desired ethyl 3-ethoxy-2-diethoxyphosphoryl-2-butenoate (**3**) as a mixture of E and Z isomers in 45:55 ratio, respectively. Distillation of the crude product yielded pure **3** in 51% yield. Friedel Crafts reaction of **3** with 3,5-dimethoxyphenol (**4**) at room temperature in the presence of trifluoromethanesulfonic acid gave 3-diethoxyphosphoryl-5,7-dimethoxy-4-methylchromen-2-one (**5**) in 70% yield. In the next step **5** was used as a Michael acceptor in the reaction with benzylmagnesium bromide in the presence of catalytic amount of CuI, yielding trans-4-benzyl-5,7-dimethoxy-4-methyl-3-diethoxyphosphoryl-3,4-dihydro-2*H*-chroman-2-one (trans-**6**) in 58% yield. Finally, the treatment of **6** with NaH and then with paraformaldehyde gave, after purification by column chromatography, target methylidenechroman-2-one (DL-247) in 89% yield.

### 2.2. Biology

Coumarins and α-methylidene-δ-lactones represent promising scaffolds in medicinal chemistry, with potential applications in cancer therapy. In this report the cytotoxicity and anticancer activity of a new synthetic hybrid DL-247 with above mentioned motifs were tested in HL-60 cells.

#### 2.2.1. Metabolic Activity and Cell Proliferation

The cytotoxic effect of DL-247 and PTL, for comparison, was studied using the MTT assay. HL-60 cells were incubated for 24 h with increasing concentrations of the tested compounds. Both compounds were highly cytotoxic and inhibited metabolic activity of the cells in a dose-dependent manner (Figure 1A). The IC_50_ value (the concentration of a drug necessary to inhibit metabolic activity of 50% of cells) for DL-247 was 1.15 ± 0.06 μM and this compound was 4 fold more active then PTL (Table 1). The cytotoxicity of PTL was almost identical for cancer and normal cells, whereas DL-247 was about 2.5 fold more toxic for HL-60, as compared with HUVEC cells.

**Figure 2:**
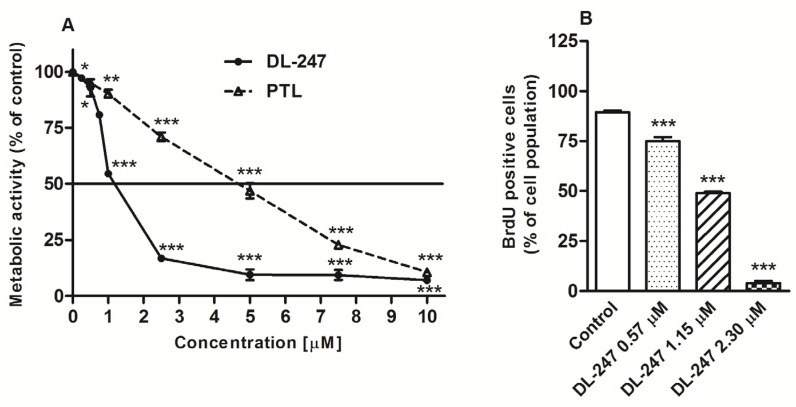
(**A**) Metabolic activity of HL-60 cells treated with various concentrations of 4-benzyl-5,7-dimethoxy-4-methyl-3-methylidene-3,4-dihydro-2*H*-chroman-2-one (DL-247) or parthenolide (PTL) for 24 h, analyzed by MTT assay. (**B**) Incorporation of bromodeoxyuridine (BrdU) into HL-60 cells treated with increasing concentrations of DL-247 for 24 h. After treatment with tested compound, BrdU was added and cells were incubated for 8 h. Then, cells were stained with PerCP-Cy™5.5 Mouse Anti-BrdU antibodies and analyzed by flow cytometry. (**A**,**B**): Data are presented as mean ± SEM of three independent experiments. Statistical significance was assessed using one-way ANOVA and a post-hoc multiple comparison Student–Newman–Keuls test. * *p* < 0.05, ** *p* < 0.01, *** *p* < 0.001, vs. control.

To establish actively cycling cell populations, HL-60 cells were incubated with bromodeoxyuridine (BrdU), an analog of thymidine. The cells treated with DL-247 (at 0.57, 1.15, and 2.30 µM concentrations) showed a dose-dependent decrease in BrdU incorporation (1.2-, 1.8-, and 22.6-fold, respectively) (Figure 2B).

#### 2.2.2. Induction of Apoptosis

Apoptosis or programmed cell death plays an essential role in homeostasis and in elimination of damaged cells [22]. It is a genetically regulated biological process, guided by the ratio of pro-apoptotic and anti-apoptotic proteins. The dysregulation or inhibition of apoptosis results in uncontrolled cell proliferation and cancer development. Thus, many therapeutic agents are designed to target apoptotic and cell cycle pathways [23,24].

**Figure 3:**
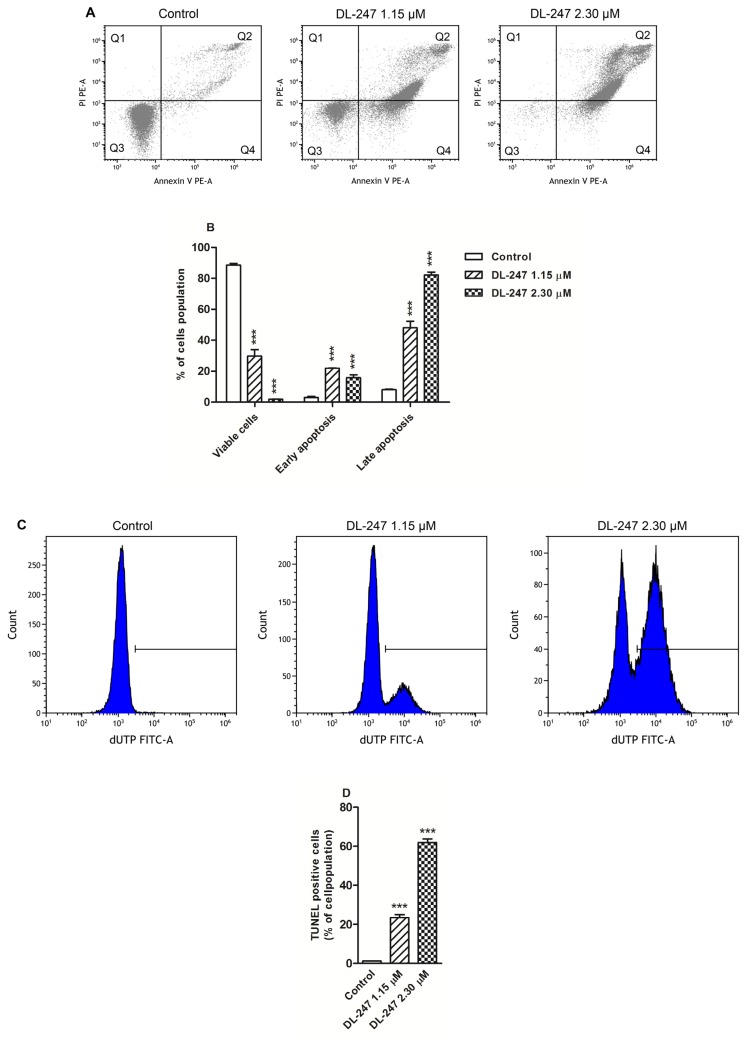
Induction of apoptosis in HL-60 cells treated with DL-247 for 24 h. (**A**) Representative scattered blots of flow cytometry analysis of apoptosis by double-staining with Annexin-V and PI. The percentage of viable cells is shown in quadrant Q3. Quadrant Q4 indicates the percentage of early apoptotic cells (Annexin V-positive cells), whereas quadrant Q2 shows the percentage of late apoptotic/death cells (Annexin V-and PI-positive cells). (**B**) Quantitative analysis of apoptotic cell death by Annexin V and PI assay. (**C**) Representative histograms of flow cytometry analysis of DNA fragmentation measured by TUNEL assay. (**D**) Quantitative analysis of DNA fragmentation. (**B**,**D**): Data are presented as mean ± SEM of three independent experiments. Statistical significance was assessed using one-way ANOVA and a post-hoc multiple comparison Student–Newman–Keuls test. *** *p* < 0.001 vs. control.

To check whether the cytotoxic effect of DL-247 in HL-60 cells was associated with apoptosis induction, the proapoptotic activity of this compound was investigated. The double-staining with Annexin V and propidium iodide (PI) was performed to evaluate the loss of plasma membrane asymmetry (phosphatidylserine externalization), which is a marker of earlier stages of apoptosis. The flow cytometry analysis revealed that DL-247 (1.15 µM) significantly increased early and late apoptotic cell fractions, up to 21.9% and 48.0% of cell population, respectively. At higher concentration (2.30 µM), a slight enhancement of the early apoptotic cells (15.8%) and a strong increase of the late apoptotic cells (82.1%) was observed (Figure 3A,B).

The ability of DL-247 to induce DNA fragmentation was then examined. DNA cleavage is the result of endonuclease activation and occurs at later stages of apoptosis. As the DNA cleavage exposes free 3′-OH termini, they can be detected by terminal deoxynucleotidyl transferase-mediated dUTP nick end labelling (TUNEL) assay. Incubation of HL-60 cells with DL-247 (at 1.15 and 2.30 µM) for 24 h led to a significant increase in the number of TUNEL positive (apoptotic) cells, whose population raised from 1.3% for control to 23.5% and 61.9%, respectively (Figure 3C,D).

#### 2.2.3. Activation of Caspases

Apoptosis regulation is triggered by the activation of caspases, a group of cysteine proteases that can cleave many cellular substrates in order to dismantle cell contents [25]. The activity of caspase-8 is required for extrinsic, whereas caspase-9 is involved in the intrinsic pathway. Following activation of the initiator caspase-8 or -9, the executioner caspase-3 cleaves a number of vital proteins leading to cell death [26,27].

**Figure 4:**
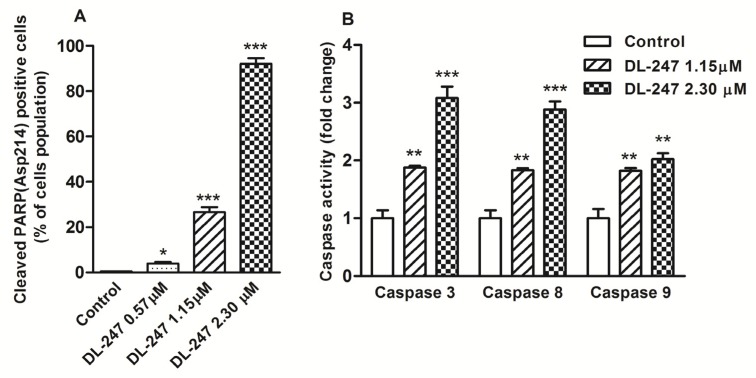
Induction of caspase activity in HL-60 cells treated with DL-247 for 24 h. (**A**) Percentage of cleaved PARP positive cells measured by flow cytometry (PE Mouse Anti-Cleaved PARP (Asp214) antibody staining). (**B**) Effect of DL-247 on caspase-3, -8 and -9 activity measured by fluorometric assay. (**A**,**B**): Data are presented as mean ± SEM of three independent experiments. Statistical significance was assessed using one-way ANOVA and a post-hoc multiple comparison Student–Newman–Keuls test. * *p* < 0.05, ** *p* < 0.01, *** *p* < 0.001, vs. control.

The activity of caspase-3 was assessed by flow cytometry using fluorochrome-labeled antibodies recognizing 89 kDa-cleaved PARP fragment, released from PARP by caspase-3 in the executive stage of apoptosis. Caspase-3 activity was detected, respectively, in 26.6% and 92.0% of the cells exposed for 24 h to DL-247 at 1.15 µM and 2.30 µM concentrations (Figure 4A).

The activity of all three caspases was confirmed by the fluorometric assay, in which fluorogenic indicators for caspase-3, -8, and -9 were used. Upon caspase cleavage, three distinct fluorophores were released, inducing the increase of fluorescence. The activity of all three caspases was enhanced, indicating that both intrinsic and extrinsic apoptotic pathways were involved in DL-247 apoptotic effect in HL-60 cells (Figure 4B).

#### 2.2.4. Involvement of the Intrinsic Pathway of Apoptosis

The intrinsic or mitochondrial pathway can be triggered by a plethora of non-receptor-mediated stimuli, including DNA damage, chemotherapeutic agents, serum starvation, and UV radiation [22]. All of these stimuli influence the integrity of the mitochondria structure and function, resulting in the loss of the mitochondrial transmembrane potential and the release of various cell death modulators, including cytochrome c, and apoptosis-inducing factor (AIF) [28,29]. Cytochrome c binds and activates Apaf-1, as well as procaspase-9, forming an “apoptosome” and leading to caspase-9 activation [30].

**Figure 5:**
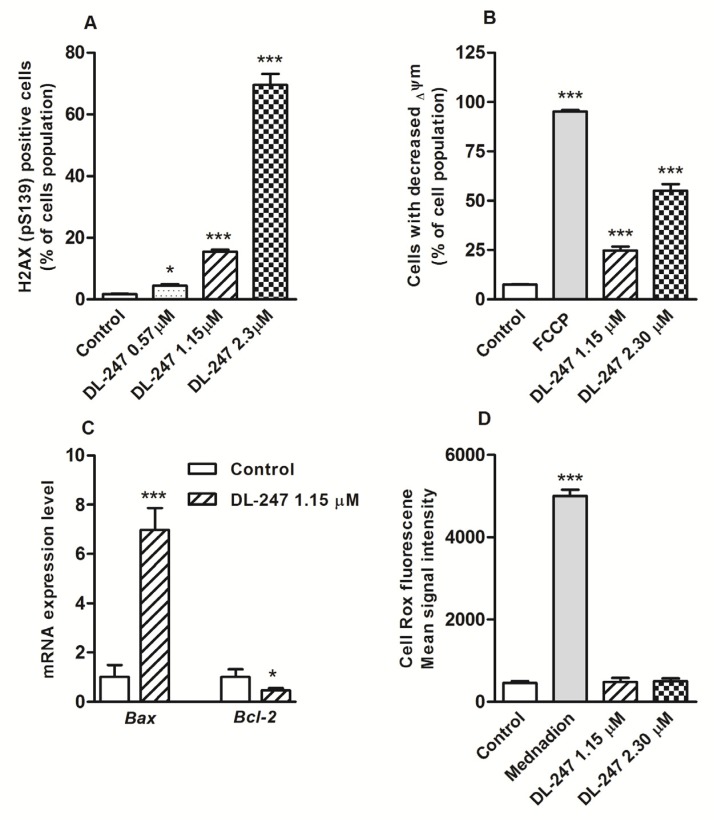
Analysis of typical features of intrinsic apoptosis pathway in HL-60 cells treated with DL-247 for 24 h. (**A**) Percentage of phosphorylated H2AX positive cells measured by flow cytometry (Alexa Fluor 647 Mouse Anti-H2AX (pS139) antibody staining). (**B**) Mitochondrial membrane potential (ΔΨm) changes measured by flow cytometry (JC-1 staining). FCCP (30 μM, 30 min incubation at 37 °C) was used as a positive control. (**C**) Bax and Bcl-2 gene expression changes analyzed by real-time PCR. (**D**) Intracellular ROS generation measured by flow cytometry (CellROX Green Reagent staining). Menadione (200 µM, 1 h incubation at 37 °C) was used as a positive control. (**A**–**D**): Data are presented as mean ± SEM of three independent experiments. (**A**,**B**,**D**): Statistical significance was assessed using one-way ANOVA and a post-hoc multiple comparison Student–Newman–Keuls test. * *p* < 0.05, *** *p* < 0.001 vs. control. C: Statistical significance was assessed using Student-t test. * *p* < 0.05, *** *p* < 0.001 vs. control.

Apoptosis induced by many anti-cancer drugs may be a consequence of DNA damage [31]. Chemical genotoxins that target DNA can inhibit DNA replication, which leads to collapse of replication forks and DNA double strand breaks (DSBs) formation. When DSBs occur, H2AX is rapidly phosphorylated at serine 139 by ataxia telangiectasia mutated (ATM), leading to the recruitment of DNA damage repair proteins at the site of damage. Phosphorylated H2AX, which promotes DNA repair and plays a role in genomic stability, can be used as a marker of DNA damage. Using flow cytometry analysis and anti-H2AX (pS139) antibodies the ability of DL-247 to generate DNA DSBs was evaluated. The treatment of HL-60 cells with DL-247 increased the levels of phosphorylated H2AX by 8.6- and 38.8-fold (for 1.15 µM and 2.30 µM, respectively), indicating that the tested compound dose-dependently inducted DNA damage (Figure 5A).

The mitochondrial membrane potential (Ψm) changes in DL-247-treated cells were analyzed by flow cytometry. FCCP served as a positive control, and as expected, produced dissipation of ΔΨm in more than 99% of cells. DL-247 caused 24.8% and 55% loss of ΔΨm at 1.15 µM and 2.30 µM concentration, respectively (Figure 5B).

The changes in the expression of two genes coding for the pro-apoptotic protein Bax and anti-apoptotic protein Bcl-2, which are involved in the regulation of mitochondrial permeability, were also studied. Bax forms pores in the mitochondrial outer membrane which leads to the release of cytochrome c and to the cell death [32]. In contrast, Bcl-2 prevents apoptosis by inhibiting the activity of Bax [33]. Therefore, the balance between these two proteins determines the cellular fate.

The exposure of HL-60 cells to DL-247 (1.15 µM) significantly up-regulated *Bax* and down-regulated *Bcl-2* gene expression (Figure 5C). The observed imbalance in the *Bax*/*Bcl-2* mRNA ratio (15:1), confirmed the promotion of apoptotic response through the mitochondrial pathway.

ROS generation can be associated with activation of both, the intrinsic and extrinsic pathways of apoptosis and poses an important mechanism of anticancer activity of numerous compounds [34,35]. To study whether DL-247-induced apoptosis was related to the presence of ROS, the intracellular ROS generation was examined using the fluorescent probe CellROX Green Reagent. Menadione, used as a positive control, caused a 11-fold enhancement of ROS production, while DL-247 did not significantly change the level of ROS at either of the tested concentrations (1.15 µM and 2.30 µM) (Figure 5D). These results indicate that DL-247-induced apoptosis was not associated with ROS generation.

#### 2.2.5. Involvement of the Extrinsic Pathway of Apoptosis

The extrinsic or death receptor pathway is initiated by death ligands such as FasL, tumor-necrosis factor α (TNFα) or TNF-related apoptosis inducing ligand (TRAIL), binding to cell surface receptors, resulting in recruitment of the adaptor protein, Fas associated death domain (FADD) and the activation of caspase-8 [36]. Active caspase-8 can either trigger effector caspases-3 directly or engage the intrinsic pathway via a cleaved form of the BH3-only protein Bid (tBid) [24].

**Figure 6:**
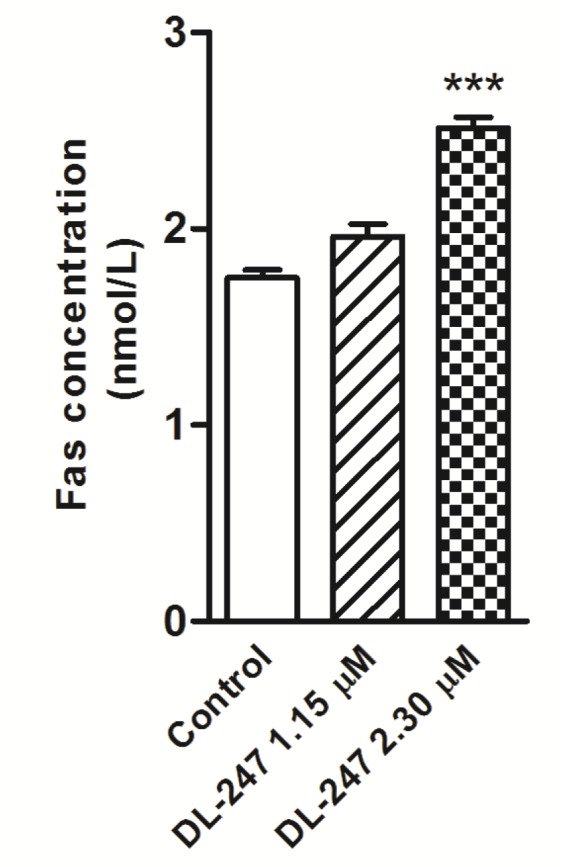
Changes in Fas concentration in HL-60 cells treated with DL-247 for 24 h analyzed by Human Fas ELISA kit. Data are presented as mean ± SEM of three independent experiments. Statistical significance was assessed using one-way ANOVA and a post-hoc multiple comparison Student–Newman–Keuls test. *** *p* < 0.001 vs. control.

Concentration of the Fas receptor (FasR) in the cell lysates obtained after 24 h-treatment of HL-60 cells with DL-247 was determined using the ELISA assay. DL-247 used at 1.15 µM concentration only slightly elevated Fas protein level. Higher concentration of this compound (2.30 µM) induced 1.4-fold increase of FasR (Figure 6). The enhancement of Fas and activation of caspase-8 and-3 (Figure 4) suggested the induction of the death receptor mediated pathway. 

#### 2.2.6. Synergistic Effects of DL-247 and Anticancer Drugs

Nowadays a treatment modality that combines two or more therapeutic agents, is a cornerstone of cancer therapy [37]. The use of drugs aimed at different targets in the cell increases the therapeutic effects compared to the mono-therapy approach and reduces the likelihood of drug resistance development [38,39]. The need for novel strategies with high-grade anticancer specificity prompts the researchers to examine new substances that, in combination with already known antineoplastic agents, could enhance response to chemotherapy.

**Figure 7:**
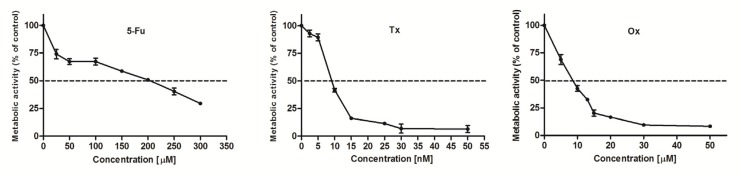
Metabolic activity of HL-60 cells treated with various concentrations of 5-Fu, Tx, or Ox for 24 h, analyzed by MTT assay.

In the current study, the influence of DL-247 on the anticancer properties of 5-FU [40], Tx [41] and Ox [42], three drugs with different mechanisms of action (antimetabolite, mitotic inhibitor, and alkylating agent, respectively) was examined. These drugs, in combined experiments, were used at IC_50_ concentration each (200 µM for 5-FU, 9 nM for Tx and 8.5 µM for Ox, determined in the MTT assay) (Figure 7).

**Figure 8:**
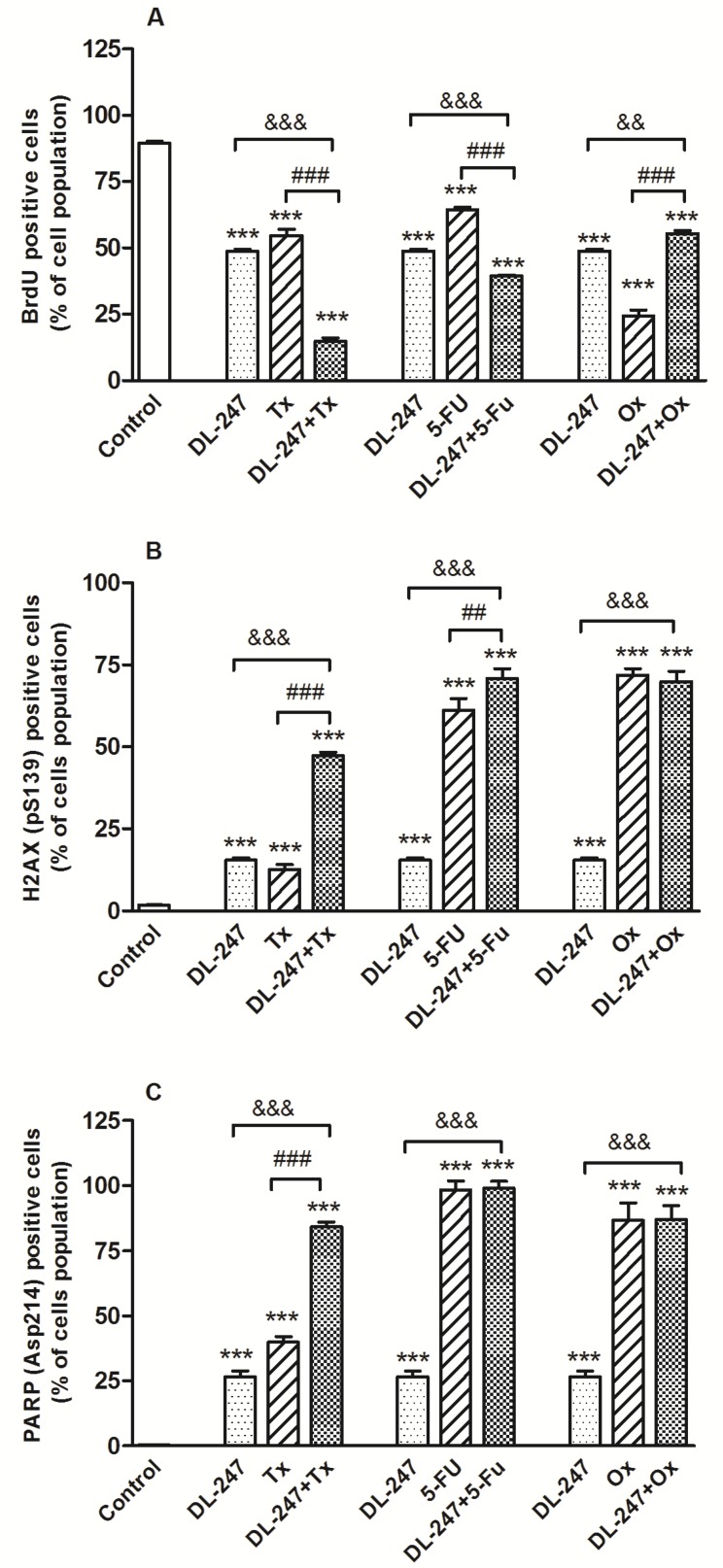
Combined effect of DL-247 (1.15 µM) and 5-FU (200 µM), Tx (9 nM) or Ox (8.5 µM) on (**A**) inhibition of cell proliferation (BrdU test), (**B**) induction of DNA damage (H2AX test) and (**C**) induction of apoptosis (PAPR test) in HL-60 cells. Cells were co-incubated with DL-247 and an anticancer drug for 24 h. Then, BrdU was added to the culture medium, and cells were incubated for additional 8 h. The cells were stained with fluorochrome-labeled anti-BrdU, anti-cleaved PARP (Asp214) and anti-H2AX (pS139) antibodies for 20 min at room temperature and analyzed by flow cytometry. (**A**–**C**): Data are presented as mean ± SEM of three independent experiments. Statistical significance was assessed using one-way ANOVA and a post hoc multiple comparison Student–Newman–Keuls test. *** *p* < 0.001 vs. control; ^&&^ *p* < 0.01, ^&&&^ *p* < 0.001 vs. DL-247; ^##^ *p* < 0.01, ^###^ *p* < 0.001 vs. respective anticancer drug.

The influence of DL-247 co-incubated with 5-FU, Tx or Ox on cell proliferation, DNA damage and apoptotic cell death was assessed by flow cytometry and fluorochrome-labeled antibodies. The combination treatments were compared with the effects caused by the drugs alone (Figure 8A–C).

**Figure 9:**
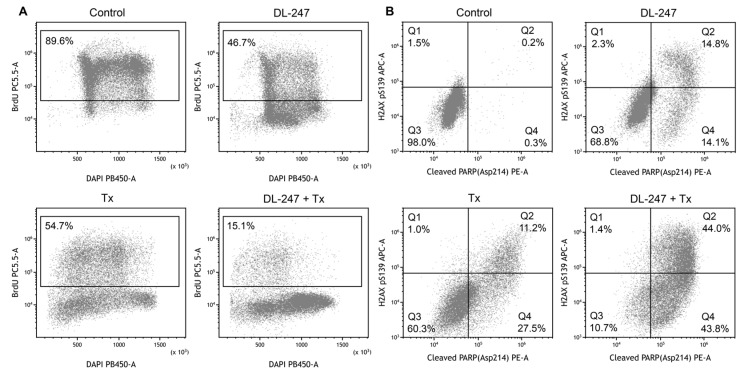
Multiparameter analysis (proliferation, DNA damage and apoptosis) of the synergistic effect of Tx (9 nM) in combination with DL-247 (1.15 µM) in HL-60 cells. Panel (**A**): DAPI vs. BrdU PerCP-Cy™5.5 staining profile. BrdU positive cells are in the inside frame. Panel (**B**): cleaved PARP (Asp214) PE vs. H2AX (pS139) Alexa profile. Squares Q1 + Q2 represent H2AX (pS139) Alexa positive cells, squares Q2 + Q4 represent Cleaved PARP (Asp214) PE positive cells, whereas square Q3 represents H2AX (pS139) Alexa and Cleaved PARP (Asp214) PE negative cells.

The co-incubation of Tx with DL-247 significantly inhibited cell proliferation and decreased the number of BrdU–positive cells (proliferating cells) form 89.6% for control to 14.8% (Figure 8A). Analysis of H2AX phosphorylation, induced by DSBs, revealed that DL-247 produced a significant synergistic effect with Tx, causing a 3.7-fold enhancement of the DNA damage in comparison to Tx alone (Figure 8B). Similar tendency was observed in relation to apoptosis. The presence of the cleaved PARP-positive cells (apoptotic cells) was detected in 39.9% of cell population for Tx alone and in 84.2% for Tx + DL-247, indicating that such combination increased the susceptibility of HL-60 cells for apoptosis (Figure 8C). The representative multiparameter flow cytometry analysis for combination of Tx and DL-247 is presented in Figure 9.

The cell proliferation and DNA damage were enhanced in the cells treated with 5-FU in combination with DL-247 (Figure 8A,B). The number of apoptotic cells induced by 5-FU alone (98.2% of cell populations) was not increased in combined treatment with DL-247 (Figure 8C).

Surprisingly the treatment of HL-60 cells with Ox and DL-247 did not reduce but enhanced cell proliferation (Figure 8A). Ox used alone was able to induce DNA damage and increase caspase-3 activity in about 71.9% and 86.7% of cell population, respectively. These high values were not further increased in the combined experiments indicating that DL-247 did not complemented the action of Ox (Figure 8B,C).

Synergy was determined using the Bliss Independence Model [43]. In this model the observed combination effect expressed as a probability (0 ≤ E_AB_ ≤ 1) can be compared to the expected additive effect given by the common formula for probabilistic independence E_A_ + E_B_(1 − E_A_) = E_A_ + E_B_ − E_A_E_B_, where 0 ≤ E_A_ ≤ 1 and 0 ≤ E_B_ ≤ 1. The Combination Indexes, calculated as: Cl = (*E*_A_+ *E*_B_ − (*E*_A_*E*_B_))/*E_AB_*, are shown in Table 2. Out of three tested anticancer drugs only Tx showed the synergistic effect in combination with DL-247 (Cl < 1).

## 3. Discussion

Acute myeloid leukemia (AML) makes about 1/3rd of all adult leukemia cases [44] and the main treatment option is chemotherapy or combination chemotherapy which is simultaneous administration of more than one anticancer drug [45]. Chemotherapy medications may have different mechanisms of action and therefore target several processes in cancer growth at the same time, increasing the chance of eliminating cancer. In the 1970s it was demonstrated that in case of acute lymphocytic leukemia [46] and Hodgkin’s lymphoma [47] combination chemotherapy could be more effective than using a single drug or two drugs one at a time, in sequence. While single-drug chemotherapy may still be better for some cancers, in the last decade combination of chemotherapy medications has been adopted for the treatment of many cancer types and has greatly increased survival rates [48,49,50]. The benefits of using combination chemotherapy rather than single agents include decreased occurrence of resistance and sometimes (but not always) reduced intensity of side effects due to the lower doses of two drugs instead of a high dose of one drug. Some drug combinations do not simply produce an additive effect in treatment, but can instead be synergistic which means that their total effect is greater than the sum of the individual effects of each drug [51].

In AML, monotherapies have not resulted in durable remissions and the improved overall survival of patients in the last years is the consequence of synergistic combination of daunorubicin and cytarabine [52]. However, optimal drug combinations are still being sought and may improve the therapeutic outcomes.

In this report the molecular mechanism of action of a novel hybrid molecule DL-247, which was selected from the series of previously described compounds combining a chroman-2-one skeleton and an α-methylidene-δ-lactone moiety was investigated [21]. DL-247 was highly cytotoxic for HL-60 cells and about 2.5 less cytotoxic for normal HUVEC cells, used for comparison. DL-247 inhibited proliferation and induced apoptosis through both intrinsic and extrinsic pathways. This compound strongly induced DNA DSBs, as evidenced by the formation of phosphorylated H2AX. DNA damage can occur through different mechanisms [31]. It is well recognized that ROS generation can increase oxidative DNA damage and lead cells to apoptosis [34,35]. However, the obtained results demonstrated that DL-247-induced apoptosis in HL-60 cells was not associated with oxidative stress, since this compound did not display pro-oxidant activity. Anticancer agents can affect cellular proliferation by increasing replication stress [53]. Alterations in the coordinated replication process typically result in the accumulation of stalled, asymmetric or broken replication forks [54]. On the other hand, the defective activation of pathways that repair DNA lesions also triggers to apoptosis. Recently, Costantino et al. [55] postulated that sesquiterpene lactones (SLs) may affect some aspects of DNA damage response (DDR). They showed that treatment of cancer cells with SLs caused accumulation of DNA lesions that triggered activation of ATM and downstream markers of DDR response. The apoptosis induced by SLs might be preceded by a failure of cells with high levels of damaged DNA to progress normally through the cell cycle. Similar results were presented in our previous study, in which incubation of MCF-7 cells with a synthetic coumarin analog led to DNA damage followed by the activation of ATM, ataxia telangiectasia and Rad3-related (ATR) and p53 proteins and down-regulation of several DNA repair-associated genes, resulting in the DNA repair defects and apoptosis [20]. We can assume that apoptosis induced by DL-247 might be a consequence of replication stress and improper functioning of the DDR system. 

The possible synergistic effects of DL-247 used in combination with the well-known anticancer drugs: 5-FU, Tx, and Ox was than studied but positive results were obtained only for co-incubation of DL-247 with Tx. Tx is a mitotic inhibitor that stabilizes microtubules and as a result, interferes with their normal breakdown during cell division. Dalton et al. have shown that agents that induce mitotic arrest may provoke DNA damage [56]. 

Despite the numerous side effects, Tx is still used as a medication of choice in the treatment of several types of cancer but in monotherapy the development of resistance is often a limiting factor. In the in vitro studies combination of Tx with an antidiabetic agent metformin was shown to increase apoptosis in HL-60 cells [57]. So far, there is not many examples of the synergistic activity of compounds containing α,β-unsaturated carbonyl moiety used in combination with anticancer drugs. The synergistic anticancer effects of PTL, an α-methylidene-γ-lactone, administrated with Tx in the in vitro studies and on some animal models were demonstrated [58]. For example, PTL increased Tx-induced apoptosis by inhibition of NF-κB binding and down-regulation of manganese superoxide dismutase (Mn-SOD) expression [59]. In the study of Sweeney et al. [60] PTL was shown to increase sensitivity of breast cancer cells (MDA-MB-231 and HBL-100) to Tx by decreasing the level of NF-κB and inducing JNK. Moreover, the combination of these two drugs enhanced survival and reduced metastasis in a xenograft model of breast cancer. Zhang et al. [61] investigated the co-administration of Tx and PTL in non-small cell lung cancer (NSCLC) in vitro and in vivo. In the co-treatment experiments PTL enhanced the inhibitory effects of Tx on cell viability in two main NSCLC cell lines, A549 and NCl-H446. The simultaneous induction of the intrinsic apoptotic pathway by PTL and the caspase-independent death pathway by Tx resulted in synergistic cytotoxicity. The efficacy of the Tx/PTL co-administration was further confirmed in the in vivo study on human A549 cell line xenografts in nude mice. Combined treatment showed a greater suppression of tumor growth relative to Tx alone, whereas PTL alone was unable to significantly inhibit tumor growth in the A549 xenograft-bearing mice. Also, a PTL/Tx co-treatment therapy decreased angiogenesis which may further enhance the efficacy observed in vivo [61,62].

In this study, the co-incubation of Tx with DL-247 was shown to significantly inhibit cell proliferation, enhance the DNA damage and increase the susceptibility of HL-60 cells to apoptosis. The obtained results indicate that easily available synthetic hybrids of chroman-2-ones with α-methylidene-δ-lactones exhibiting strong anticancer properties are also effective chemosensitizers that can enhance apoptotic capacity of Tx in the leukemia cells.

## 4. Materials and Methods

### 4.1. Synthesis of DL-247

4-Benzyl-5,7-dimethoxy-4-methyl-3-methylidene-3,4-dihydro-2*H*-chroman-2-one (DL-247), was synthesized according to the two-step reaction sequence described in detail earlier [21].

### 4.2. Cell Culture and Treatment

Human promyelocytic leukemia cell line (HL-60) was obtained from the European Collection of Cell Cultures (ECACC, Salisbury, UK) while normal human umbilical vein endothelial (HUVEC) cells were purchased from the American Type Culture Collection (ATCC, Manassas, VA, USA). HL-60 cells were cultured in RPMI 1640 plus GlutaMax I medium (Gibco/Life Technologies, Carlsbad, CA, USA), supplemented with 10% heat-inactivated FBS (Biological Industries, Beit-Haemek, Israel) and antibiotics (100 µg/mL streptomycin and 100 U/mL penicillin) (Sigma-Aldrich, St. Louis, MO, USA). HUVEC cells were grown in EGM-2 Endothelial Medium BulletKit (Lonza, Basel, Switzerland), containing all necessary supplements for endothelial cell proliferation. Cells were maintained at 37 °C in a 5% CO_2_ atmosphere and were grown until 80% confluent.

For all experiments the tested compound was dissolved in sterile dimethylsulfoxide (DMSO) (Sigma-Aldrich, St. Louis, MO, USA) and further diluted in the culture medium. The final concentration of DMSO in the cell cultures was less than 0.1% *v/v*. Controls without and with 0.1% DMSO were performed in each experiment. At the used concentration DMSO had no effect on the observed parameters. PTL, Ox and Tx were purchased from Tocris Bioscience (Bristol, UK) and 5-FU and menadione from Sigma-Aldrich (St. Louis, MO, USA). Ox and menadione were dissolved directly in the culture medium. PTL, 5-Fu, Tx, and FCCP were dissolved in DMSO and then diluted in culture medium, as described above.

### 4.3. Metabolic Activity Assay (MTT)

The effect of DL-247 on metabolic activity of HL-60 cells was determined by 3-(4,5-dimethylthiazol-2-yl)-2,5 diphenyl tetrazolium bromide (MTT) assay, according to the Mosmann method, as described elsewhere [21]. Cells were seeded on 96-well plates in 100 µL of culture medium and exposed to various concentrations of the tested compound. After 24 h of treatment, MTT (5 mg/mL in PBS) was added and cells were incubated for additional 2 h. The plates were centrifuged (300× *g*, 15 min, room temperature), supernatants were removed and 100 µL of DMSO was added to dissolve MTT formazon crystals. The absorbance was measured at 560 nm using an automated iMark Microplate reader (Bio-Rad, Hercules, CA, USA) and compared with control (untreated cells). The IC_50_ values were calculated from the concentration–response curves.

### 4.4. Annexin V-FITC/PI Assay

Induction of apoptosis was examined using FITC Annexin V Apoptosis Detection Kit I (BD Bioscience, San Jose, CA, USA). Briefly, the cells were seeded in the 6-well plates at the density of 2.0 × 10^5^/mL in 2 mL of cell culture medium and treated with the tested compound for 24 h. Then, the cells were collected by centrifugation (300× *g*, 5 min), washed with PBS, re-suspended in the binding buffer and stained with FITC-conjugated annexin V and propidium iodide (PI). The samples were analyzed by flow cytometry using CytoFLEX (Beckman Coulter, Inc, Brea, CA, USA). Data analysis was performed using Kaluza Analysis Software v2 (Beckman Coulter, Inc, Indianapolis, IN, USA).

### 4.5. Tunel Assay

DNA fragmentation was analyzed by the terminal deoxynucleotidyl transferase mediated dUTP nick end labeling (TUNEL) assay, using APO-DIRECT™ kit (BD Biosciences Pharmingen), followed by flow cytometry analysis. Briefly, the cells were seeded in the 6-well plates at the density of 2.0 × 10^6^ cells/mL in 2 mL of the culture medium and treated with DL-247 or DMSO (for control). After 24 h incubation, the cells were collected by centrifugation (300× *g*, 5 min), washed with PSB and fixed in 1% (*w/v*) paraformaldehyde in PBS. The cells were rinsed with PBS, re-suspended in 70% (*v/v*) ice cold ethanol and stored at −20 °C for at least 12 h. After washing, the cells were stained with FITC dUTP in the presence of TdT enzyme for 60 min at 37 °C and analyzed in PI/RNase Staining Buffer by flow cytometry using CytoFLEX (Beckman Coulter, Inc, Brea, CA, USA).

### 4.6. Caspase 3, Caspase 8, and Caspase 9 Activity

Caspase-3, -8, and -9 activities were measured using a fluorometric Caspase 3, Caspase 8, and Caspase 9 Multiplex Activity Assay Kit (Abcam, Cambridge, UK). This kit provides DEVD-ProRed, IETD-R110 and LEHD-AMC as fluorogenic indicators for caspase 3, caspase 8 and caspase 9 activity, respectively. Upon caspase cleavage, three distinct fluorophores are released: ProRed™ (red fluorescence), R110 (green fluorescence) and AMC (blue fluorescence). Briefly, cells were seeded in the 96-well plates at the density of 2 × 10^5^ cells/well in 90 μL of the culture medium and treated with DL-247 for 24 h. Then, the fluorogenic indicators for each caspase were added to the cells. After 50 min incubation the fluorescence intensity was read (Ex/Em = 535/620 nm, 490/525 nm and 370/450 nm for caspase 3, -8, and -9, respectively), using FlexStation 3 Multi-Mode Microplate Reader (Molecular Devices, CA, USA). The results were expressed as an increase of caspase-3, -8, and -9 activities relative to the control.

### 4.7. Mitochondrial Membrane Potential Changes

Changes in the mitochondrial membrane potential (∆Ψm) were assessed by flow cytometry using the BD™ MitoScreen Kit (BD Bioscience, San Jose, CA, USA), containing a cationic lipophilic JC-1 dye. Briefly, the cells were seeded in the 6-well plates at the density of 2.0 × 10^5^/mL in 2 mL of the cell culture medium and treated with the tested compound for 24 h. The cells were collected by centrifugation (300× *g*, 5 min), washed with PBS and stained with JC-1 for 15 min at 37 °C, in the dark. Then, cells were washed, re-suspended in assay buffer and analyzed by flow cytometry using CytoFLEX (Beckman Coulter, Inc, Brea, CA, USA). A mitochondrial uncoupler, carbonyl cyanide-4-(trifluoromethoxy)phenylhydrazone (FCCP, Sigma-Aldrich, Louis, MO, USA) was used as a positive control (30 µM, 30 min treatment at 37 °C).

### 4.8. Determination of Reactive Oxygen Species (ROS)

The intracellular production of reactive oxygen species (ROS) was evaluated by flow cytometry analysis using cell-permeable CellROX Green Reagent (Life Technologies, Carlsbad, CA, USA). Briefly, the cells were seeded in the 24-well plates at a density 2.0 × 10^5^/mL in 1 mL of the cell culture medium and treated with DL-247 or DMSO (control) for 24 h. Then, CellROX Green Reagent (final concentration 5 µM) was added to each well and the cells were incubated for 30 min at 37 °C, in the dark. The cells were centrifuged (300× *g*, 5 min), washed with PBS, re-suspended in PBS and analyzed by flow cytometry using CytoFLEX (Beckman Coulter, Inc, Brea, CA, USA). A redox-cycling agent, menadione (Sigma Aldrich, Louis, MO, USA) was used as a positive control (100 µM, 1 h treatment at 37 °C).

### 4.9. Real-Time PCR

The mRNA levels of *Bax* and *Bcl-2* were assessed using quantitative real-time PCR. Briefly, HL-60 cells were seeded in the 6-well plates at a density 2.0 × 10^5^/mL in 2 mL of cell culture medium and treated DL-247 for 24 h. The cells were washed twice with PBS and the total RNA was isolated using Total RNA Mini Kit (A&A Biotechnology, Gdynia, Poland), according to manufacturer’s protocol. cDNA was synthesized using the Enhanced Avian HS RT-PCR Kit and oligo(dT)_12-18_ primers (Sigma-Aldrich, St. Louis, MO, USA). The amplification was performed using Brilliant II SYBR Green QPCR Master Mix (Agilent Technologies, Inc. Santa Clara, CA, USA) and gene specific primers (Table 3) in Mx3005P QPCR System (Agilent Technologies, Inc. Santa Clara, CA, USA) according to the manufacturer’s guidelines. Glyceraldehyde 3-phosphate dehydrogenase (GAPDH) was used as a reference gene. The expression levels of the tested genes were determined by the 2^-∆∆CT^.

### 4.10. Human Factor-Related Apoptosis (FAS) Detection by ELISA Assay

Evaluation of the level of FAS protein was performed using Human Factor-related Apoptosis ELISA Kit (BT Lab Shanghai Korain Biotech Co, Shanghai, China) according to the manufacturer’s protocol. Briefly, the cells were seeded in 25 cm^2^ culture flasks at a density of 7.0 × 10^5^ cells/mL in 10 mL of the culture medium and treated with DL-247 for 24 h. The cells were collected by centrifugation (300× *g*, 5 min), washed with PBS and re-suspended in ice-cold PBS containing protease inhibitor cocktail (Sigma-Aldrich, Louis, MO, USA). The cells were incubated on ice for 10 min and damaged by sonication. The cell lysates were centrifuged (5000× *g* for 10 min at 4 °C) and the supernatants were aliquoted and stored at −20 °C. Protein concentration was estimated using Bradford protein assay (from Bio-Rad Laboratories Inc., CA, USA). The samples were added to a FAS antibody pre-coated plate and the biotinylated human FAS primary antibody and streptavidin-HRP secondary antibody were added. After incubation with the substrate solution, the stop solution was added and the absorbance was measured at 450 nm using FlexStation 3 Multi-Mode Microplate Reader (Molecular Devices, LLC). The concentration of FAS was calculated using the professional Curve Expert 1.3 software (Hyams Development).

### 4.11. Analysis of Cell Proliferation, Apoptosis, and DNA Damage by Flow Cytometry

The analysis of cell proliferation, DNA damage and apoptotic cell death was performed using the “Apoptosis, DNA Damage, and Cell Proliferation Kit” (BD Bioscience, San Jose, CA, USA), according to the manufacturer’s protocol. Briefly, the cells were seeded in the 6-well plates at a density of 2.0 × 10^5^ cells/mL in 2 mL of the culture medium and treated with DL-247 for 24 h. The bromodeoxyuridine (BrdU, 10 μM) was added and the cells were incubated for additional 8 h. Next, the cells were centrifuged (300× *g*, 5 min), counted, fixed and permeabilized according to the manufacturer’s instructions. The cells were incubated with DNase (300 µg/mL in PBS) for 1 h at 37 °C and then simultaneously stained with fluorochrome-labeled anti-BrdU, H2AX (pS139), and cleaved PARP (Asp214) antibodies, for 20 min at room tepmerature. After washing, DNA staining with DAPI (1 µg/mL of the staining buffer) was performed and the cells were analyzed by flow cytometry using CytoFLEX (Beckman Coulter, Inc, Brea, CA, USA).

### 4.12. Statistical Analysis

All statistical calculations were performed using Prism 5.0 software (GraphPad Software Inc., San Diego, CA, USA). Results from three independent experiments performed in triplicate were expressed as mean ± SEM. Statistical significance was assessed using Student’s *t*-test (for comparisons of two treatment groups) or one-way ANOVA followed by a post-hoc multiple comparison Student–Newman–Keuls test (for comparisons of three or more groups). *P*-values < 0.05 were considered statistically significant.

## 5. Conclusions

In summary, the study demonstrated that the synthetic chroman-2-one DL-247 produced potent cytotoxic effect and inhibited proliferation in HL-60 cells. The apoptotic cell death induced by this compound was triggered by both, the mitochondrial and receptor-mediated pathways and involved activation of the initiator and executioner caspases (-8, -9, and -3).

DL-247 was shown to enhance the anticancer activity of the mitotic inhibitor Tx in HL-60 cells. In particular, the combination of DL-247 with Tx exhibited a synergistic effect on DNA damage generation, apoptosis induction and inhibition of cell proliferation.

The presented findings indicate that DL-274 has a potential to sensitize cancer cells to anticancer drugs and may be further tested in the combination experiments.

## Figures and Tables

**Table 1:** Cytotoxic activity of DL-247 and PTL on HL-60 cell line and normal HUVEC cells.

Compound	IC_50_ (µM)	
	HL-60	HUVEC
DL-247	1.15 ± 0.06	2.87 ± 0.03
PTL	4.75 ± 0.01	5.15 ± 0.12

**Table 2:** Combination index values of DL-247 and anticancer drugs on HL-60 cells.

	Inhibition of Proliferation		Induction of DNA Damage		Induction of Apoptosis	
Combination	Fa	Cl	Fa	Cl	Fa	Cl
DL-247 + Tx	0.83	**0.80**	0.47	**0.55**	0.84	**0.66**
DL-247 + 5-Fu	0.56	1.09	0.71	**0.95**	0.99	1.00
DL-247 + Ox	0.38	2.23	0.70	1.09	0.87	1.04

Fa: fraction affected, Cl: combination index. Cl > 1.0 indicates antagonism, Cl < 1.0 indicates synergism.

**Table 3:** Primer sequences for real-time PCR reaction.

Gene	Primer Sequences	
	Forward	Reverse
*Bax*	5′ ACCCGGTGCCTCAGGATGCGT 3′	5′ GGCAAAGTAGAAAAGGGCGAC 3′
*Bcl-2*	5′ CATGCTGGGGCCGTACAG 3′	5′ GAACCGGCACCTGCACAC 3′
*GAPDH*	5′ GTCGCTGTTGAAGTCAGAGGAG 3′	5′ CGTGTCAGTGGTGGACCTGAC 3′

## References

[1] Saklani A., Kutty S.K. (2008). Plant-derived compounds in clinical trials. Drug Discov. Today.

[2] Riveiro M.E., De Kimpe N., Moglioni A., Vázquez R., Monczor F., Shayo C., Davio C. (2010). Coumarins: Old compounds with novel promising therapeutic perspectives. Curr. Med. Chem..

[3] Venugopala K.N., Rashmi V., Odhav B. (2013). Review on natural coumarin lead compounds for their pharmacological activity. Biomed. Res. Int..

[4] Marshall M.E., Kervin K., Benefield C., Umerani A., Albainy-Jenei S., Zhao Q., Khazaeli M.B. (1994). Growth-inhibitory effects of coumarin (1,2-benzopyrone) and 7-hydroxycoumarin on human malignant cell lines in vitro. J. Cancer Res. Clin. Oncol..

[5] Riveiro M.E., Vazquez R., Moglioni A., Gomez N., Baldi A., Davio C., Shayo C. (2008). Biochemical mechanisms underlying the pro-apoptotic activity of 7,8-dihydroxy-4-methylcoumarin in human leukemic cells. Biochem. Pharmacol..

[6] Nasr T., Bondock S., Youns M. (2014). Anticancer activity of new coumarin substituted hydrazide-hydrazone derivatives. Eur. J. Med. Chem..

[7] Zhang S., Won Y.K., Ong C.N., Shen H.M. (2005). Anti-cancer potential of sesquiterpene lactones: Bioactivity and molecular mechanisms. Curr. Med. Chem. Anticancer Agents.

[8] Janecka A., Wyrębska A., Gach K., Fichna J., Janecki T. (2012). Natural and synthetic α-methylenelactones and α-methylenelactams with anticancer potential. Drug Discov. Today.

[9] Pati H.N., Das U., Sharma R.K., Dimmock J.R. (2007). Cytotoxic thiol alkylators. Mini Rev. Med. Chem..

[10] Mathema V.B., Koh Y.S., Thakuri B.C., Sillanpää M. (2012). Parthenolide, a sesquiterpene lactone, expresses multiple anti-cancer and anti-inflammatory activities. Inflammation.

[11] Kupchan S.M., Hemingway R.J., Werner D., Karim A., McPhail A.T., Sim G.A. (1968). Vernolepin, a novel elemanolide dilactone tumor inhibitor from *Vernonia hymenolepis*. J. Am. Chem. Soc..

[12] Weinheimer A.J., Chang C.W.J., Matson J. (1979). Naturally occurring cembranes. Fortschr. Chem. Org. Naturst..

[13] Quintana J., Estévez F. (2018). Recent Advances on Cytotoxic Sesquiterpene Lactones. Curr. Pharm. Des..

[14] Ren Y., Yu J., Kinghorn A.D. (2016). Development of Anticancer Agents from Plant-Derived Sesquiterpene Lactones. Curr. Med. Chem..

[15] Grech-Baran M., Pietrosiuk A. (2010). Arglabina–lakton seskwiterpenowy o właściwościach przeciwnowotworowych. Biul. Wydz. Farm. WUM.

[16] Modranka J., Albrecht A., Jakubowski R., Krawczyk H., Różalski M., Krajewska U., Janecka A., Wyrębska A., Różalska B., Janecki T. (2012). Synthesis and biological evaluation of α-methylidene-δ-lactones with 3,4-dihydrocoumarin skeleton. Bioorg. Med. Chem..

[17] Deredas D., Huben K., Janecka A., Długosz A., Pomorska D.K., Mirowski M., Krajewska U., Janecki T., Krawczyk H. (2016). Synthesis and anticancer properties of 3-methylene-4-(2-oxoalkyl)-3,4-dihydrocoumarins. Chem. Commun..

[18] Wyrębska A., Gach K., Lewandowska U., Szewczyk K., Hrabem E., Modranka J., Jakubowski R., Janecki T., Szymański J., Janecka A. (2013). Anticancer Activity of New Synthetic α-Methylene-δ-Lactones on Two Breast Cancer Cell Lines. Basic Clin. Pharmacol. Toxicol..

[19] Dlugosz A., Gach-Janczak K., Szymanski J., Deredas D., Krawczyk H., Janecki T., Janecka A. (2018). Anticancer Properties of a New Hybrid Analog AD-013 Combining a Coumarin Scaffold with an α-methylene-δ-lactone Motif. Anticancer Agents Med. Chem..

[20] Długosz A., Drogosz J., Deredas D., Janecki T., Janecka A. (2018). Involvement of a coumarin analog AD-013 in the DNA damage response pathways in MCF-7 cells. Mol. Biol. Rep..

[21] Jakubowski R., Pomorska D.K., Długosz A., Janecka A., Krajewska U., Różalski M., Mirowski M., Bartosik T., Janecki T. (2017). Synthesis of 4,4-Disubstituted 3-Methylidenechroman-2-ones as Potent Anticancer Agents. Chem. Med. Chem..

[22] Elmore S. (2007). Apoptosis: A review of programmed cell death. Toxicol. Pathol..

[23] Hu W., Kavanagh J.J. (2003). Anticancer therapy targeting the apoptotic pathway. Lancet Oncol..

[24] Zaman S., Wang R., Gandhi V. (2014). Targeting the apoptosis pathway in hematologic malignancies. Leuk. Lymphoma.

[25] Degterev A., Boyce M., Yuan J. (2003). A decade of caspases. Oncogene.

[26] Fischer U., Jänicke R.U., Schulze-Osthoff K. (2003). Many cuts to ruin: A comprehensive update of caspase substrates. Cell Death Differ..

[27] Fulda S., Debatin K.M. (2006). Extrinsic versus intrinsic apoptosis pathways in anticancer chemotherapy. Oncogene.

[28] Saelens X., Festjens N., Vande Walle L., van Gurp M., van Loo G., Vandenabeele P. (2004). Toxic proteins released from mitochondria in cell death. Oncogene.

[29] Wang C., Youle R.J. (2009). The role of mitochondria in apoptosis. Annu. Rev. Genet..

[30] Chinnaiyan A.M. (1999). The apoptosome: Heart and soul of the cell death machine. Neoplasia.

[31] Roos W.P., Kaina B. (2006). DNA damage-induced cell death by apoptosis. Trends Mol. Med..

[32] Westphal D., Dewson G., Czabotar P.E., Kluck R.M. (2011). Molecular biology of Bax and Bak activation and action. Biochim. Biophys. Acta.

[33] Yip K.W., Reed J.C. (2008). Bcl-2 family proteins and cancer. Oncogene.

[34] Valko M., Leibfritz D., Moncol J., Cronin M.T., Mazur M., Telser J. (2007). Free radicals and antioxidants in normal physiological functions and human disease. Int. J. Biochem. Cell Biol..

[35] Schumacker P.T. (2006). Reactive oxygen species in cancer cells: Live by the sword, die by the sword. Cancer Cell.

[36] Thorburn A. (2004). Death receptor-induced cell killing. Cell. Signal..

[37] Bayat Mokhtari R., Homayouni T.S., Baluch N., Morgatskaya E., Kumar S., Das B., Yeger H. (2017). Combination therapy in combating cancer. Oncotarget.

[38] Housman G., Byler S., Heerboth S., Lapinska K., Longacre M., Snyder N., Sarkar S. (2014). Drug resistance in cancer: An overview. Cancers.

[39] Li F., Zhao C., Wang L. (2014). Molecular-targeted agents combination therapy for cancer: Developments and potentials. Int. J. Cancer.

[40] Longley D.B., Harkin D.P., Johnston P.G. (2003). 5-Fluorouracil: Mechanisms of action and clinical strategies. Nat. Rev. Cancer.

[41] Weaver B.A. (2014). How Taxol/paclitaxel kills cancer cells. Mol. Biol. Cell.

[42] Alcindor T., Beauger N. (2011). Oxaliplatin: A review in the era of molecularly targeted therapy. Curr. Oncol..

[43] Greco W.R., Bravo G., Parsons J.C. (1995). The search for synergy: A critical review from a response surface perspective. Pharmacol. Rev..

[44] Estey E., Döhner H. (2006). Acute myeloid leukaemia. Lancet.

[45] DeVita V.T., Chu E. (2008). A history of cancer chemotherapy. Cancer Res..

[46] Pinkel D., Hernandez K., Borella L., Holton C., Aur R., Samoy G., Pratt C. (1971). Drug dose and remission duration in childhood lymphocytic leukemia. Cancer.

[47] DeVita V.T., Serpick A.A., Carbone P.P. (1970). Combination chemotherapy in the treatment of advanced Hodgkin’s disease. Ann. Intern. Med..

[48] Choi J.H., Choi Y.W., Kang S.Y., Jeong G.S., Lee H.W., Jeong S.H., Park J.S., Ahn M.S., Sheen S.S. (2020). Combination versus single-agent as palliative chemotherapy for gastric cancer. BMC Cancer.

[49] Bashraheel S.S., Domling A., Goda S.K. (2020). Update on targeted cancer therapies, single or in combination, and their fine tuning for precision medicine. BioMed. Pharmacother..

[50] Qiang H., Chang Q., Xu J., Qian J., Zhang Y., Lei Y., Han B., Chu T. (2020). New advances in antiangiogenic combination therapeutic strategies for advanced non-small cell lung cancer. J. Cancer Res. Clin. Oncol..

[51] Hu M., Huang P., Wang Y., Su Y., Zhou L., Zhu X., Yan D. (2015). Synergistic Combination Chemotherapy of Camptothecin and Floxuridine through Self-Assembly of Amphiphilic Drug-Drug Conjugate. Bioconjug. Chem..

[52] Sallman D.A., Lancet J.E. (2017). What are the most promising new agents in acute myeloid leukemia?. Curr. Opin. Hematol..

[53] Jackson S.P., Bartek J. (2009). The DNA-damage response in human biology and disease. Nature.

[54] Gottifredi V., Prives C. (2005). The S phase checkpoint: When the crowd meets at the fork. Semin. Cell Dev. Biol..

[55] Costantino V.V., Mansilla S.F., Speroni J., Amaya C., Cuello-Carrión D., Ciocca D.R., Priestap H.A., Barbieri M.A., Gottifredi V., Lopez L.A. (2013). The sesquiterpene lactone dehydroleucodine triggers senescence and apoptosis in association with accumulation of DNA damage markers. PLoS ONE.

[56] Dalton W.B., Nandan M.O., Moore R.T., Yang V.W. (2007). Human cancer cells commonly acquire DNA damage during mitotic arrest. Cancer Res..

[57] Asik A., Kayabasi C., Ozmen Yelken B., Yılmaz Susluer S., Dogan Sigva Z.O., Balcı Okcanoglu T., Saydam G., Biray Avci C., Gunduz C. (2018). Antileukemic effect of paclitaxel in combination with metformin in HL-60 cell line. Gene.

[58] Wyrębska A., Gach K., Janecka A. (2014). Combined effect of parthenolide and various anti-cancer drugs or anticancer candidate substances on malignant cells in vitro and in vivo. Mini Rev. Med. Chem..

[59] Patel N.M., Nozaki S., Shortle N.H., Bhat-Nakshatri P., Newton T.R., Rice S., Gelfanov V., Boswell S.H., Goulet R.J., Sledge G.W. (2000). Paclitaxel sensitivity of breast cancer cells with constitutively active NF-kappaB is enhanced by IkappaBalpha super-repressor and parthenolide. Oncogene.

[60] Sweeney C.J., Mehrotra S., Sadaria M.R., Kumar S., Shortle N.H., Roman Y., Sheridan C., Campbell R.A., Murry D.J., Badve S. (2005). The sesquiterpene lactone parthenolide in combination with docetaxel reduces metastasis and improves survival in a xenograft model of breast cancer. Mol. Cancer Ther..

[61] Zhang D., Qiu L., Jin X., Guo Z., Guo C. (2009). Nuclear factor-kappaB inhibition by parthenolide potentiates the efficacy of Taxol in non-small cell lung cancer in vitro and in vivo. Mol. Cancer Res..

[62] Gao Z.W., Zhang D.L., Guo C.B. (2010). Paclitaxel efficacy is increased by parthenolide via nuclear factor-kappaB pathways in in vitro and in vivo human non-small cell lung cancer models. Curr. Cancer Drug Targets.

